# Dr. Harrison's Letter, on Medical Reform

**Published:** 1809-10

**Authors:** E Harrison


### ( CIRCULAR. )

*London, September 23, 1809.*

SIR,

I Have the honour to forward to you the enclosed resolutions, and hope no apology will be deemed necessary for again calling your attention to the present defective state of medical education and practice in the country, or to the advantages that will arise to the community at large from obliging future members of the profession to be properly qualified before they engage in their respective occupations.

I beg leave to inform you, that the bill, alluded to in the resolutions here annexed, was drawn up last summer by a gentleman of high legal rank, and much in the confidence of government. It has since that time been submitted to many persons, practising in the different branches of medicine, and to others not of the profession; by all of whom, I am happy to add, it has been highly approved. Though the bill had been carefully revised, no attempt was made to present it to parliament last winter, principally for want of an adequate fund to pay the fees of parliament and provide for contingencies. The pecuniary resources of the committee appointed in London to conduct the undertaking are still very deficient, and I write to request the favour of you to endeavour to induce the faculty and others of your acquaintance to assist you in procuring subscriptions from such as are favourable to the measure, whether they be in the Profession or not, that the Bill may be offered to Parliament early in the approaching Session. The names and amount of all subscriptions, with the expences incurred, will be given to the Public in a future stage of the business.

The declaration concerning the Royal Colleges of Physicians and Surgeons is founded chiefly on the opinion of Mr. Sergeant Williams, who, in answer to the Queries proposed to him at the desire of the Committee, respecting the jurisdiction of the Royal College of Physicians, observes, "The result of the whole is this: The great object cannot be attained. Any person, with a degree or without one, or with a licence from the College or without, may practise physic in England at a greater distance than seven miles from London, whether he be fit or not, without any person to controul him; otherwise than that, by the common law of England, if a person is guilty of

*mala*

*mala praxis,* whether it be for curiosity and experiment, or by neglect, he is guilty of a great misdemeanor and offence, for which he can be indicted. But he is not under the controul, or supervision, or correction of the College of Physicians, or any other; or liable to any penalty for so doing, notwithstanding the statute 14th and 15th of Henry 8th, and therefore this mischief can only be, and I conceive ought to be, remedied by the Legislature."

From want of authority in the Corporations of the united Kingdom to controul the provincial Faculty, and examine into their fitness to enter upon practice, a very large majority are now exercising the different branches of medicine, as regular practitioners, with fictitious titles or purchased diplomas, by which frauds the Public are deceived often to their utter ruin, and well educated men are cruelly deprived of their fair emoluments. Among these, several have undergone the essential parts of a liberal education, only overlooking the forms, and are worthy of all the confidence that can be reposed in them; but as they are liable to be confounded with ignorant pretenders, it will certainly be for the safety as well as the advantage of the Community, to fix an adequate restraint upon the conduct of those, to whom they commit so valuable a treasure as their health. If we may credit reports, the medical establishments of the Army, Navy, and East India Company are equally incompetent; and is it politic in us to expose unnecessarily the protectors of our independence and of our dearest interests, when, under Divine Favour, the prosperity of the Empire can only be affected by want of numbers?

It appears from the opinion cited above, that in the country the vilest Empiric is as much at liberty to exercise the curative art, as the most skilful and regular practitioner. Quacks, aware of their ignorance and misdeeds, artfully conceal them, by compounding and dispensing their own drugs. A sagacious writer, adverting to their numerous contrivances to impose upon the credulous invalid, observes, "As matters stand at present, it is easier to cheat a man out of his life than of a shilling, and almost impossible to detect or punish the fraud. In order to reduce the shoals of Empirics to some order, and place them under an impartial tribunal, it is directed in the bill, that such as cannot obtain certificates from three or more magistrates assembled in sessions, are to be immediately suppressed, and as the remainder die away, their places are to be supplied by responsible persons.

With a view to correct other abuses, it is proposed, that no unadmitted Practitioner shall be suffered to act as Physician, unless he be a graduate of some University. Surgeons must possess diplomas from a College of Surgeons. Apothecaries are to submit to a five years Apprenticeship. Accoucheurs to be Physicians, Surgeons, or Apothecaries, who have spent twelve months in the study and practice of Midwifery.

To instruction is to be added in all cases strict examinations, and the names with the titles of the Faculty are to be annually published in a Register. Such are the leading features of the proposed Bill, which differs in several particulars from the "Outline" formerly published; and it is expected that by instituting these several defences against designing intruders, Society at large will be essentially benefited, because no Person will venture hereafter to engage in medical practice, until he has obtained his authority from a respectable source, and is therefore entitled to the confidence of his Patients. Thus at the same time that the present race of medical men of every description, except empirical Pretenders, are legally confirmed in their accustomed titles and occupations, a lasting improvement will be gradually established.

I have the honor to be, Sir,
Your most obedient humble servant,
E. HARRISON.

*P. S.* I hope to be honored with an answer to this Address. To obtain the freedom from postage it must be directed as formerly, "Dr. Harrison, Horncastle," under cover to "George Harrison, Esq. Treasury, London."

It is much to be lamented that for want of a Medical Register a large majority of the provincial Faculty must necessarily be overlooked in this circular application. As the omission arises from their names or residence not being known to the writer, he trusts they will be pleased to accept this apology, and come forward in aid of the common cause as much as they would have done had they been individually solicited.

CRITICAL

